# Reliability of tibiofemoral contact area and centroid location in upright, open MRI

**DOI:** 10.1186/s12891-020-03786-1

**Published:** 2020-11-30

**Authors:** Andrew M. Schmidt, David J. Stockton, Michael A. Hunt, Andrew Yung, Bassam A. Masri, David R. Wilson

**Affiliations:** 1 Centre for Hip Health and Mobility, University of British Columbia, 7/F - 2635 Laurel Street, Robert HN Ho Research Centre, Vancouver, BC V5Z 1M9 Canada; 2 Department of Orthopaedics, University of British Columbia, Vancouver, BC Canada; 3 Clinician Investigator Program, University of British Columbia, Vancouver, BC Canada; 4 Department of Physical Therapy, University of British Columbia, Vancouver, BC Canada; 5 Motion Analysis and Biofeedback Laboratory, University of British Columbia, Vancouver, BC Canada; 6 MRI Research Center, University of British Columbia, Vancouver, BC Canada

**Keywords:** Standing MRI, Contact area, Tibiofemoral joint, Knee, Anterior cruciate ligament, Reliability, Biomechanics, MRI

## Abstract

**Background:**

Imaging cannot be performed during natural weightbearing in biomechanical studies using conventional closed-bore MRI, which has necessitated simulating weightbearing load on the joint. Upright, open MRI (UO-MRI) allows for joint imaging during natural weightbearing and may have the potential to better characterize the biomechanical effect of tibiofemoral pathology involving soft tissues. However open MRI scanners have lower field strengths than closed-bore scanners, which limits the image quality that can be obtained. Thus, there is a need to establish the reliability of measurements in upright weightbearing postures obtained using UO-MRI.

**Methods:**

Knees of five participants with prior anterior cruciate ligament (ACL) rupture were scanned standing in a 0.5 T upright open MRI scanner using a 3D DESS sequence. Manual segmentation of cartilage regions in contact was performed and centroids of these contact areas were automatically determined for the medial and lateral tibiofemoral compartments. Inter-rater, test-retest, and intra-rater reliability were determined and quantified using intra-class correlation (ICC_3,1_), standard error of measurement (SEM), and smallest detectable change with 95% confidence (SDC_95_). Accuracy was assessed by using a high-resolution 7 T MRI as a reference.

**Results:**

Contact area and centroid location reliability (inter-rater, test-retest, and intra-rater) for sagittal scans in the medial compartment had ICC_3,1_ values from 0.95–0.99 and 0.98–0.99 respectively. In the lateral compartment, contact area and centroid location reliability ICC_3,1_ values ranged from 0.83–0.91 and 0.95–1.00 respectively. The smallest detectable change in contact area was 1.28% in the medial compartment and 0.95% in the lateral compartment. Contact area and centroid location reliability for coronal scans in the medial compartment had ICC_3,1_ values from 0.90–0.98 and 0.98–1.00 respectively, and in the lateral compartment ICC_3,1_ ranged from 0.76–0.94 and 0.93–1.00 respectively. The smallest detectable change in contact area was 0.65% in the medial compartment and 1.41% in the lateral compartment. Contact area was accurate to within a mean absolute error of 11.0 mm^2^.

**Conclusions:**

Knee contact area and contact centroid location can be assessed in upright weightbearing MRI with good to excellent reliability. The lower field strength used in upright, weightbearing MRI does not compromise the reliability of tibiofemoral contact area and centroid location measures.

**Supplementary Information:**

The online version contains supplementary material available at 10.1186/s12891-020-03786-1.

## Background

Magnetic resonance (MR) has been used to assess tibiofemoral (TF) joint mechanics for a number of applications, including explaining how acute injury such as anterior cruciate ligament (ACL) rupture increases the risk for osteoarthritis (OA) [1–6]. Some measurements have been made for static postures, while others have been made dynamically, and measurements made include kinematics [3, 6, 7], cartilage contact area [3, 6, 8], and centroid location [3, 6, 7].

The strength of MR imaging is the ability to directly assess soft tissue structures such as cartilage. With the body in functional positions there is an opportunity to study the biomechanical behaviour of these structures. In biomechanical studies using conventional closed-bore MR, imaging cannot be performed during natural weightbearing. To address this, approaches include imaging cartilage in supine before and after a knee loading activity is performed [9, 10], positioning the participant supine in the scanner with an axial load applied to the foot (closed kinetic chain) [3, 6, 8], and applying a torque to the shank while the participant lies supine [7].

The reliability and accuracy for contact area and centroid location from studies with simulated loading have been estimated. The coefficient of variation (CV) for tibiofemoral contact area and centroid location, which indicates the extent of variability between multiple testing sessions, has ranged between 3.1–9.0% and 0.3–3.3%, respectively [3, 7, 8, 11]. Determining contact area by combining MRI with biplanar radiography has shown a slightly larger standard error of measurement of 14 ± 11% in a cadaveric validation study [12].

There is emerging interest in open MR machines that allow scanning to take place with participants in functional positions like standing. Similar to the utility of standing X-ray in the clinical investigation and operative planning of knee osteoarthritis, standing MRI may have the potential to better characterize the biomechanical effect of tibiofemoral pathology involving soft tissues like ligaments, menisci, and cartilage. Injury to such structures is a risk factor in the development of osteoarthritis, and UO-MRI may be useful for studying these injuries before the eventual development of deforming bony changes.

Upright, open MRI (UO-MRI) addresses the limitations of simulated weightbearing in supine scanners by allowing joint imaging during weightbearing [7, 13, 14]. However, UO-MRI scanners have a less homogenous magnetic field and a lower magnetic field strength than standard closed-bore scanners, which limits the image quality that can be obtained [15]. Thus, there is a need to establish the reliability of measurements in upright weightbearing postures obtained using UO-MRI.

The aims of this study were: 1) to assess the reliability and accuracy of tibiofemoral cartilage contact area and centroid location acquired both sagitally and coronally; and 2) to describe the implementation of a UO-MRI protocol that permits acquisition of these measures in vivo under physiologic weight-bearing conditions.

## Methods

This study was approved by the UBC Clinical Research Ethics Board (H18–01459). All participants provided informed, written consent (Additional file 2).

### Participants

A sample of 5 patients from a larger comparative cohort study volunteered for reliability analysis. The cohort study was a convenience sample of 18 patients with prior ACL rupture. Patients were recruited through posted notifications and targeted e-mails (Additional file 1). The five patients selected for this study were the only patients from the larger cohort who consented to complete the scanning process, a three-hour procedure, on two separate dates, thus allowing for test-retest reliability analysis.

Inclusion criteria for the cohort study were: 1) adult participants between the ages of 18–50 years old with unilateral, isolated ACL ruptures; 2) intact cartilage and evidence of complete ACL rupture on MRI; 3) reported ACL rupture within the last 5 years and if reconstructed, done within 1 year from injury; and 4) have completed a full rehabilitation program and returned to regular sport or recreational activities.

Exclusion criteria were: 1) associated ligament rupture other than the ACL (though incomplete MCL ruptures were not excluded); 2) known knee osteoarthritis diagnosed by a physician; 3) presence of other joint disease; 4) incompletely rehabilitated injury, defined as a range of motion less than 0–130 degrees, quadriceps atrophy, or persistent mechanical symptoms; 5) individuals prohibited from undergoing MRI based on the MRI screening form (Additional file 3); 6) history of fainting, or evidence of change in orthostatic blood pressure; 7) prior or subsequent knee surgery other than diagnostic arthroscopy; 8) history of corticosteroid injection to either knee; and 9) bilateral ACL rupture or ACL re-rupture.

Demographic data from participants were collected including age, height, body mass, date of injury, time from injury to surgery (if applicable), and time from injury to study participation.

### Imaging

Participants were scanned standing in a 0.5 T upright, open MRI (MROpen, Paramed, Genoa, Italy). All scans were done in the morning, participants were instructed not to do any impact exercise prior to scanning, and participants were seated for 30 min prior to scanning, during which time questionnaires were administered. Participants wore compression socks to minimize venous pooling in the lower extremities during standing scans. Participants then stood for 15 min prior to acquiring standing scans to ensure a cartilage deformation equilibrium had been reached. Each participant wore a chest harness suspended from an aluminum ceiling track safety-rated to 450 lbs. (Handicare, Concord, ON) as a precautionary measure in case the participant fainted during upright scanning. No weight was borne through the bars or the harness. Standing scans of the ACL-injured leg were acquired with the knees in full extension, with the participant instructed to stand comfortably and distribute their weight equally between legs. Three support bars (shins, buttocks, and hands) were placed to help the participant remain still during scanning. We obtained sagittal and coronal images with a double echo steady state T2 sequence (Table 1) using a commercial 2-channel knee coil (ParaMed) suspended around the knee. The sequence was optimized to provide excellent cartilage signal quickly enough to minimize the effects of patient movement and fatigue while standing. The data were denoised using an optimized blockwise nonlocal means denoising filter [16], and the component DESS images were subsequently fit to a signal model with a global T1 estimate of 0.5 [17].

**Table 1:** Imaging parameters used for UO-MRI scan and for the high-resolution 7 T MRI scan

	0.5 T UO-MRI	7 T MRI
Pulse sequence	3D DESS	2D multi-slice RARE
Repetition time (ms)	16	2200
Echo time (ms)	6	8.4
Field of view (cm)	22 × 22 × 16	6 × 6
Acquisition matrix size	256 × 256 × 38 (zero filled to 256 × 256 × 64)^a^	256 × 256, 50 slices
Slice thickness (μm)	2500	35.0
Slice gap (μm)	0	0
Voxel dimensions (μm)	859 × 859 × 2500	23.4 × 23.4 × 35.0
Flip angle (°)	30	180
Bandwidth (Hz/pixel)	146.9	318.4
Total scan time (min)	3 min 30s	28 min 10s

^a^Note the voxel dimensions are interpolated in the slab direction

**Fig. 1:**
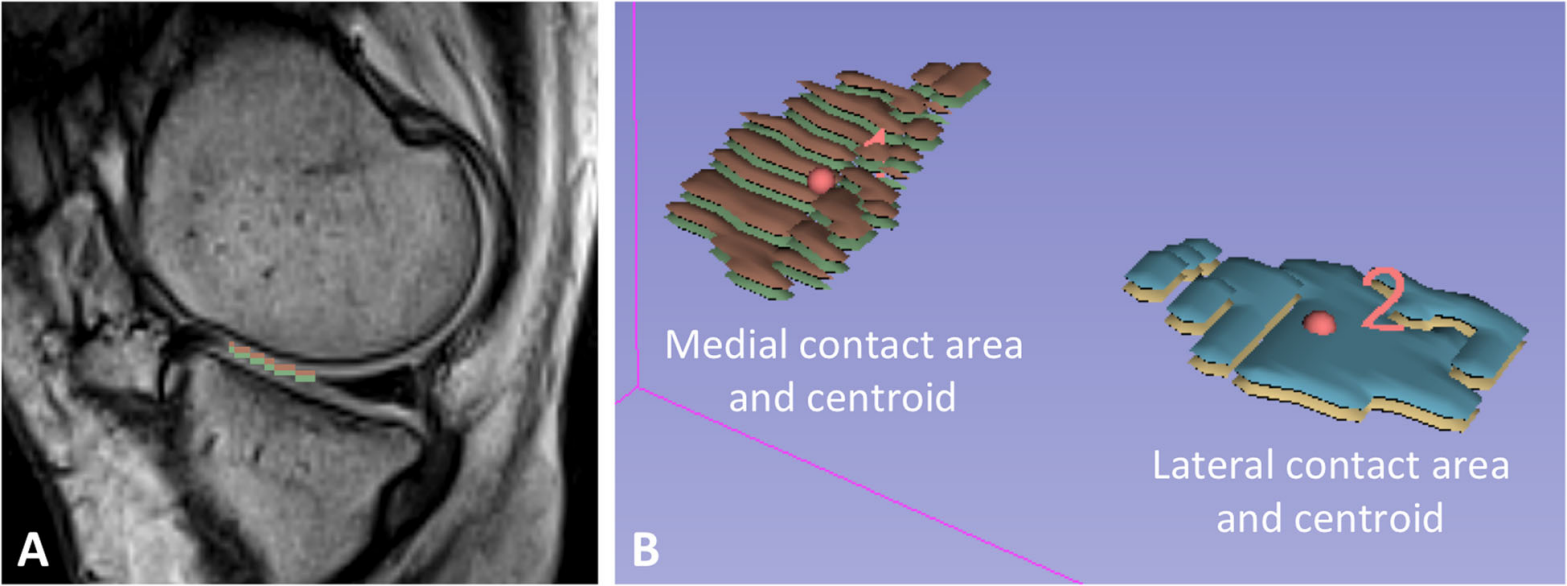
**a** Representative sagittal slice from the medial compartment of a participant showing the tibial cartilage in contact (green) and the femoral cartilage in contact (brown). **b** Representative volumes of medial and lateral contact areas and contact centroids

Two trained raters, A.M.S. and D.J.S, with 2 years and 3 years’ experience respectively, performed segmentation for all data sets. Both raters were trained in knee joint segmentation by a post-doctoral fellow with 10 years of experience in segmenting MSK data. Prior to the study both raters established a set of general guidelines for segmentation. All data sets were anonymized, and a numerical code was assigned to each patient. Raters identified tibiofemoral contact regions by manually tracing regions with no visible separation between cartilage surfaces on each image slice using the Editor module in 3D Slicer [18] (http://www.slicer.org) in both the coronal and sagittal planes (Fig. 1a). Raters selected voxels of cartilage that were in direct contact and did not contain any contribution from other structures (e.g. *meniscus* or synovial fluid). Volumes were created that represented medial and lateral contact areas, each with a known number of voxels (Fig. 1b). We multiplied the number of voxels in contact by their axial dimensions (length and width) to calculate contact areas for the medial and lateral compartments. To account for differences in size between subjects, the cartilage contact area measurement in the axial plane was normalized by taking the ratio (%) of the contact area over the maximum axial cross-sectional area of the tibial plateau.

The centroid location was calculated as the geometric center generated from the contact area segmentations in the medial and lateral compartments (Fig. 1b). A validated joint coordinate system was employed to locate contact area centroids within a consistent coordinate frame [19, 20]. Centroid location was reported in mm and also quantified as a percentage on the tibial plateau in the medial (0%) to lateral (100%) and posterior (0%) to anterior (100%) directions to account for differences in size between participants. The coordinate system was based on the bony landmarks and axis definitions of an established joint coordinate system convention [19]. Reference bony landmarks were established from supine scout scans of the hip, knee, and ankle, with the scan position relative to each other noted from the difference in UO-MRI scan table position. Positions and orientations of the coordinate systems in the upright posture were determined by registering supine images of the tibia and femur to corresponding upright images using Analyze 12.0 (AnalyzeDirect, Inc., Overland Park, KS). The test-retest reliability of using this joint coordinate system in the UO-MRI was assessed in a previous study [20], and ICC reliability values ranged from 0.95–0.99 for joint rotations and translations.

### Accuracy

We assessed the accuracy of contact area measurement by comparing our method in the UO-MRI to reference measurements of contact area made in a 7 T MR scanner (Bruker Biospin, Ettlingen, Germany) for two cartilage preparations at two load levels. We created two cartilage contact preparations by dissecting a bovine knee and extracting medial and lateral tibial and femoral blocks using a handsaw. The block dimensions were approximately 30 mm by 30 mm in the anteroposterior direction and mediolateral direction and were approximately 20 mm in the axial (compressive) direction. The blocks were oriented on polycarbonate tissue mounts in a manner that maximized contact of the flattest part of the mating joint surfaces. The bony side of each osteochondral block was affixed to the tissue mounts with cyanoacrylate glue. The preparations were immersed in phosphate-buffered saline and positioned in an MR-compatible compression chamber such that axial compression could be applied by rotating a Delrin plunger (2 mm thread) within the capsule of the compression chamber. The samples were positioned such that opposing cartilage surfaces were touching but not compressed, and images were acquired. An axial load was then applied until cartilage compression could be visualized, and the specimen was re-scanned. Five minutes were allowed to pass in between cartilage compression and re-scanning in order to permit the cartilage to equilibrate. The displacement of the plunger was marked on the outside of the chamber so that the process could be repeated. On completion, the load was removed, and the cartilage was given time to re-equilibrate. The process was performed first in the UO-MRI and then in the 7 T MRI, using imaging parameters listed in Table 1. In a previous study, intra-observer repeatability of segmentation of loaded tibial and femoral cartilage images in this 7 T scanner was within 2.3 and 3.3 voxels for cartilage depth, 95% of the time [21].

### Statistics

Inter-rater, test-retest, and intra-rater reliability statistics were calculated for tibiofemoral contact area and centroid location. Inter-rater reliability was obtained for two raters who individually segmented and calculated contact areas for each scan. Test-retest reliability was established by scanning each participant twice, with approximately 1 month between scans, with one rater (D. J. S.) segmenting both scans. Intra-rater reliability was obtained for one rater (A. M. S.) segmenting the contact areas for each sample 3 times, each 2 weeks apart. We calculated the intra-class correlation coefficient for fixed raters (ICC_3,1_) using the methods described by Shrout and Fleiss [22], as well as the standard error of measurement (SEM), and the smallest detectable change with 95% confidence (SDC_95_). ICCs less than 0.5 indicated poor reliability; 0.5 to 0.75 moderate reliability; 0.75 to 0.9 good reliability; and greater than 0.9 excellent reliability. All metrics were obtained for both coronal and sagittal scans.

We assessed contact area accuracy by finding the mean absolute error (MAE) for contact areas measured using UO-MRI and those measured for the same region and load using 7 T MRI from images obtained in the sagittal plane.

## Results

Descriptive characteristics for the 5 participants included in the reliability analysis are reported in Table 2. There were 4 female participants and 1 male; 3 had undergone ACL reconstruction and 2 had not.

**Table 2:** Descriptive characteristics of participants in reliability analysis

	Mean (SD)
Age (years)	23.4 (4.2)
Time since injury (years)	2.9 (1.8)
BMI (kg/m^2^)	23.3 (1.1)

Mean absolute contact areas were 452 mm^2^ (±103) and 314 mm^2^ (±41) for medial and lateral compartments, respectively. Mean normalized contact areas were 13.7% (±2.6) and 9.7% (±1.6) for medial and lateral compartments, respectively.

**Fig. 2:**
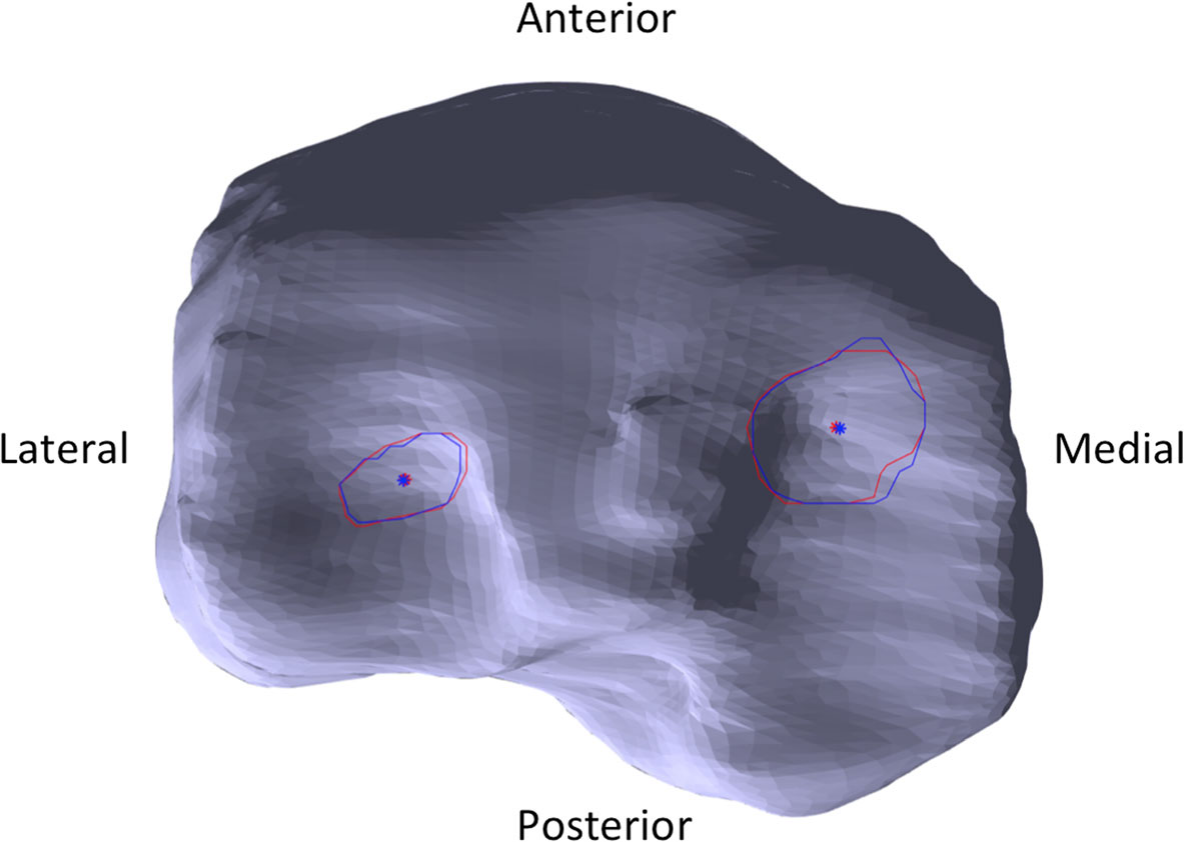
Axial view of a standardized tibial plateau with representative cartilage contact areas and centroid locations. Rater one cartilage contact area and centroids are in red and rater two cartilage contact area and centroids are in blue. The SEM for these measurements is 0.22 mm and 0.72 mm for the X and Y components of the medial centroid; 0.66 mm and 0.28 mm for the X and Y components of the lateral centroid, and 3.22 mm^2^ and 1.08 mm^2^ for the medial and lateral contact areas

For scans acquired in the sagittal plane, contact area ICC_3,1_ values (including inter-rater, test-retest, and intra-rater reliability) ranged from 0.94 to 0.99 in the medial compartment, and 0.83 to 0.91 in the lateral compartment (Table 3). From the test-retest data, contact area SDC_95_ was 1.28% in the medial compartment and 0.95% in the lateral compartment. Qualitatively, contact regions were very similar between raters (Fig. 2), and centroid location demonstrated high reliability (Table 4). SDC_95_ for medial centroid locations in the X and Y direction were 3.39 and 4.94% (1.89 mm and 2.29 mm), respectively. SDC_95_ for lateral centroid locations in the X and Y direction were 4.41 and 3.85% (3.31 mm and 1.42 mm), respectively.

**Table 3:** Contact area reliability for sagittal UO-MRI scans

	Medial compartment				Lateral compartment			
	ICC_3,1_ (95%CI)	*P*-Value	SEM (%)	SEM (mm^2^)	ICC_3,1_ (95%CI)	*P*-Value	SEM (%)	SEM (mm^2^)
Inter-Rater	0.95 (0.59–0.99)	0.002	0.39	16.77	0.83 (0.06–0.98)	0.021	0.44	15.48
Test-Retest	0.94 (0.56–0.99)	0.002	0.46	13.33	0.84 (0.10–0.98)	0.017	0.34	11.40
Intra-Rater	0.99 (0.94–1.00)	< 0.001	0.21	6.49	0.91 (0.64–0.99)	< 0.001	0.31	7.92

**Table 4:** Centroid location reliability for sagittal UO-MRI scans

	Medial compartment						Lateral compartment					
	ICC_3,1_ (95%CI)	*P*-Value	X SEM (%)	Y SEM (%)	X SEM (mm)	Y SEM (mm)	ICC_3,1_ (95%CI)	*P*-Value	X SEM (%)	Y SEM (%)	X SEM (mm)	Y SEM (mm)
Inter-Rater	0.99 (0.97–1.00)	< 0.001	0.71	1.62	0.44	0.64	0.95 (0.83–0.99)	< 0.001	0.95	2.81	0.57	1.10
Test-Retest	0.94 (0.56–0.99)	< 0.001	1.22	1.78	0.68	0.83	0.98 (0.91–0.99)	< 0.001	1.59	1.39	1.19	0.51
Intra-Rater	0.99 (0.94–1.00)	< 0.001	0.15	2.44	0.14	0.76	1.00 (0.99–1.00)	< 0.001	0.34	0.57	0.18	0.23

For scans acquired in the coronal plane, contact area ICC_3,1_ (including inter-rater, test-retest, and intra-rater reliability) ranged from 0.90 to 0.97 in the medial compartment and 0.76 to 0.94 in the lateral compartment (Table 5). From the test-retest data, SDC_95_ was 0.65% in the medial compartment and 1.41% in the lateral compartment. Again, centroid location demonstrated high reliability (Table 6). SDC_95_ for medial centroid locations in the X and Y direction were 4.04 and 6.22% (2.63 mm and 2.96 mm), respectively. SDC_95_ for lateral centroid locations in the X and Y direction were 3.38 and 9.83% (2.30 mm and 4.40 mm), respectively.

**Table 5:** Contact area reliability for coronal UO-MRI scans

	Medial compartment				Lateral compartment			
	ICC_3,1_ (95%CI)	*P*-Value	SEM (%)	SEM (mm^2^)	ICC_3,1_ (95%CI)	*P*-Value	SEM (%)	SEM (mm^2^)
Inter-Rater	0.90 (0.35–0.99)	0.007	0.54	18.92	0.87 (0.19–0.99)	0.013	0.34	13.12
Test-Retest	0.98 (0.86–1.00)	< 0.001	0.23	6.02	0.76 (−0.14–0.97)	0.041	0.51	14.19
Intra-Rater	0.97 (0.85–1.00)	< 0.001	0.35	9.20	0.94 (0.74–0.99)	< 0.001	0.23	6.47

**Table 6:** Centroid location reliability for coronal UO-MRI scans

	Medial compartment						Lateral compartment					
	ICC_3,1_ (95%CI)	*P*-Value	X SEM (%)	Y SEM (%)	X SEM (mm)	Y SEM (mm)	ICC_3,1_ (95%CI)	*P*-Value	X SEM (%)	Y SEM (%)	X SEM (mm)	Y SEM (mm)
Inter-Rater	0.99 (0.98–1.00)	< 0.001	0.29	1.50	0.31	0.49	0.99 (0.95–1.00)	< 0.001	0.71	1.43	0.59	0.62
Test-Retest	0.94 (0.92–0.99)	< 0.001	1.46	2.24	0.95	1.07	0.93 (0.74–0.98)	< 0.001	1.22	3.55	0.79	1.59
Intra-Rater	1.00 (1.00–1.00)	<0.001	0.27	0.54	0.18	0.20	1.00 (0.99–1.00)	<0.001	0.23	0.66	0.11	0.22

In the accuracy analysis, data from one sample (medial compartment unloaded) was discarded due to a technical error. During scanning of this sample at the 7 T MRI, the field of view did not include the full bovine specimen, and thus did not include the full contact area of the sample. Unfortunately, this error was discovered during image post-processing after scanning had concluded. The remaining areas obtained in the UO-MRI for the lateral compartment unloaded, medial compartment loaded, and lateral compartment loaded were: 120 mm^2^, 271 mm^2^, and 254 mm^2^, respectively. Corresponding areas measured using the 7 T MRI were 126 mm^2^, 258 mm^2^, and 240 mm^2^, respectively. The mean absolute error was 11 mm^2^.

## Discussion

We assessed in vivo inter-rater, test-retest, and intra-rater reliability of tibiofemoral contact area and centroid location measurements for UO-MRI scans in both sagittal and coronal planes. We evaluated the accuracy of our contact area measurements by comparing measurements made using the UO-MRI to measurements made in a 7 T MRI scanner for a bovine knee model. All measures of contact area reliability, including inter-rater, test-retest, and intra-rater, ranged from good to excellent for coronal and sagittal scans. Qualitatively, there was close correspondence between contact regions identified by different readers (Fig. 2). The accuracy analysis found an overall mean absolute error of 11 mm^2^ between areas found from 7 T MRI and from the UO-MRI. Our results suggest that sagittal or coronal scans are similarly well-suited to evaluate cartilage contact and centroid location in the tibiofemoral joint, with slightly higher repeatability values resulting from sagittal plane acquisition and evaluation.

Our reporting of SDC_95_ provides useful information for designing future research studies that complements the more widely-used ICC values. SDC_95_ indicates the smallest amount of change that provides 95% confidence that a true change has occurred and is not due to inherent measurement error. Our finding of SDC_95_ of 2–2.5 mm for changes in contact location in sagittal plane images is smaller than a 4.2 mm difference reported between knees with ACL rupture and healthy knees estimated using a biplanar radiography/MRI image registration approach [2]. This suggests that our UO-MRI approach can effectively detect differences in centroid location due to ACL deficiency. Similarly, previous investigations have estimated that ACL injury changes tibiofemoral contact area by as much as 94.8 mm^2^ medially and 56.3 mm^2^ laterally [23]. Our largest estimated SEM was 18.9 mm^2^, corresponding to a SDC95 of 52.4 mm^2^, indicating that UO-MRI may also be effective at detecting contact area differences.

Our measures of contact area and centroid location reliability in UO-MR are comparable to those from 3 T conventional closed-bore scans despite using a lower resolution scanner. For inter-rater reliability, our finding of contact area ICC in the medial compartment of 0.95 is consistent with findings in 3 T MRI (0.90) [3]. Our finding of contact area ICC in the lateral compartment for inter-rater reliability (0.83) was also in the good range for an ICC value, although it had a much wider confidence interval range (0.06 to 0.98) and was lower than findings in 3 T MRI (0.92) [3]. The inter-rater contact location ICCs (0.99 medially and 0.95 laterally) were also similar to those found in 3 T (0.99 medially and 0.91 laterally) [3]. For intra-rater reliability our findings for contact area ICC were 0.99 medially and 0.91 laterally, which was again consistent with 3 T MRI findings of 0.97 both medially and laterally [3]. Our intra-rater contact location ICCs (0.99 medially and 0.98 laterally) were similar to those found in 3 T (1.00 medially and 0.91 laterally) [3]. It should be noted that all participants in our study had a prior ACL rupture, which is not true for these previous studies. No previous study has evaluated the test-retest reliability of contact area and centroid location in vivo, although one cadaveric study examined the patellofemoral joint using a 1.5 T magnet and found a test-retest ICC value of 0.98, which is comparable to our results [24]. The slightly higher variation in test-retest reliability in the current study is likely due to slight differences in participant posture and positioning between test dates, which may be easier to control in a cadaveric study. The test-retest reliability measures will be of value in experimental design, especially for studies requiring testing on more than 1 day. Our accuracy results, which found a mean absolute error of 11.0 mm^2^, suggest higher accuracy for our method than the results from a cadaver study using a silicone casting technique reference standard, which found a standard error of measurement of 14% [12]. This may be because the reference method of the current study (high field MRI) is different from the reference method for the previous study (silicone casting). The absolute values of our contact areas were slightly higher than previously reported values [6, 23], though the ratio of lateral to medial contact area were similar. This may have been partially due to cartilage creep, since our participants stood in a weightbearing position for 15 min prior to standing. Additionally, differences in tibiofemoral contact area in the same knees have been observed depending on the MRI sequence used [8]. The T2 DESS sequence that we used greatly enhances cartilage, which may have accounted for our high contact area values.

The primary strength of this study is that it provides a comprehensive assessment of the role of repeated scans and the intra- and inter-individual differences in raters on the reliability of tibiofemoral contact measures. The good to excellent reliability results are supported by an accuracy assessment. Incorporation of both sagittal and coronal plane assessment and reporting of SDC_95_ may be useful in protocol development for future studies. Given the advantages for ecological validity with the UO-MRI approach for these assessments compared to traditional supine MRI, we feel that our findings have important implications for the study of knee joint mechanics and function in future UO-MRI studies.

The findings should be considered in light of some limitations. First, reliability was assessed in ACL-ruptured knees only. The cartilage of these participants may not be representative of cartilage in uninjured knee joints. While the effect of this on our findings is not clear, we performed the scans relatively soon after injury and it is unlikely that enough time had passed for cartilage degeneration to significantly affect our segmentation process. Second, the accuracy assessment used a small number of samples. Because it was an ex vivo study, it did not include the effects of subject movement or blood flow. The sample was also immersed in phosphate buffered saline, which can increase SNR and may have influenced our results. Additionally, the bovine osteochondral blocks used may not have adequately represented human tibiofemoral contact behaviour. We chose 7 T MRI as a reference measure because it allowed the highest resolution images possible while still allowing the same loading rig and loads to be used. The lengthy scan time and cost of the 7 T scanner hindered our ability to process more samples for accuracy assessment; similarly, we were not able to establish the reliability of measuring contact area in the 7 T MRI before we used it as the reference standard. Third, the low resolution of the UO-MRI due to the low magnetic field strength may have reduced variability, and the lower field homogeneity of the UO-MRI may have been a source of error and may present issues for future test-retest reliability measurements. This should be taken into account when planning future experiments. Finally, some of our intra-class correlation results were bounded by wide confidence intervals, in particular test-retest measures of lateral contact area. These results should be considered in the interpretation of lateral contact area measures using the current methods. More raters and tests would improve the certainty of our estimates and further development of area measurement methods will be required if more reliable measures of lateral contact area are needed.

## Conclusions

We found that, in spite of lower field strength and homogeneity, UO-MRI can be used to measure tibiofemoral contact area and centroid location with comparable reliability to higher field closed-bore scanners and sufficient reliability to detect differences in contact area and centroid location due to ACL injury. These findings support using UO-MRI for direct measurements of tibiofemoral contact area in standing, which has potential to improve our understanding of normal and pathological knee biomechanics.

## Supplementary Information

**Additional file 1.**

**Additional file 2.**

**Additional file 3.**

**Additional file 4.**

## Data Availability

The datasets generated and/or analysed during the current study are included in this published article and its supplementary information files.

## Abbreviations

UO-MRI: Upright, Open MRI
MC: Medial tibiofemoral compartment
LC: Lateral tibiofemoral compartment
ICC_3,1_: Intra-class correlation
SEM: Standard error of measurement
SDC_95_: Smallest detectable change with 95% confidence
MAE: Mean absolute error
MR: Magnetic resonance
ACL: Anterior cruciate ligament
OA: Osteoarthritis
CV: Coefficient of variation
SD: Standard deviation
BMI: Body mass index
