# Supplementary material for: Reliability of tibiofemoral contact area and centroid location in upright, open MRI
Source: BMC Musculoskelet Disord. 2020 Nov 30;21:795. doi: 10.1186/s12891-020-03786-1 (PMC7702694; doi:10.1186/s12891-020-03786-1)
Supplement: Supplementary file 1 — Additional file 1. [file 12891_2020_3786_MOESM1_ESM.pdf]

Vancouver CoastalHealth
Promoting wellness. Ensuring care.

UBC

CENTRE for HIP Health and Mobility

# Have you torn your ACL before?

## Would you like to be a part of a research study?

## We are looking for participants who wish to undergo an MRI in a new Upright, Open MRI scanner

We are looking for adult patients age 18-50 years old who have torn their ACL, to be a part of a study that seeks to quantify the cartilage changes that occur after this injury using a new, upright, open MRI scanner (UO-MRI). If you have torn only your ACL (on one side) within the last 10 years, and have fully rehabilitated from your injury, regardless of whether or not you had a surgery to reconstruct the ACL, this study may be of interest to you.

The study involves have a series of MRI scans at the UO-MRI scanner, located in the Blackmore Pavilion at Vancouver General Hospital. These scans are for research purposes only; they are not clinical diagnostic MRI scans. Participation will take up to 3 hours on a single day.

If you are interested, please contact us. We ask that you come in for a short screening session to ensure that you qualify for the study.

## Participants will be compensated $100.

## If you are interested, or would like more information, please contact:


Dr. David Stockton, on behalf of Principal Investigator Dr. Dave Wilson

[redacted]

## Letter of Initial Contact – Upright Open MRI

### Title: Quantification of cartilage changes using upright, open Magnetic Resonance Imaging (UO-MRI) after Anterior Cruciate Ligament (ACL) injury

**Dear Patient,**

We are writing to inform you of a study involving patients who have ruptured their ACL.

**Principal Investigator:** Dr. Dave Wilson, DPhil, Professor in the Department of Orthopaedics and Co-Director of the Centre for Hip Health and Mobility, UBC

**Co-Investigators:** Dr. Pierre Guy, MD, MBA, FRCSC, Head of Orthopaedic Trauma at Vancouver General Hospital and Associate Professor in the Department of Orthopaedics, UBC; Dr. Bas Masri, MD, FRCSC, Professor and Head of the Department of Orthopaedics, UBC; Dr. David Stockton, MD; Andrew Yung, MSc; Dr. Jane Desrochers, PhD; Andrew Schmidt, BSc; & Dr. Honglin Zhang, PhD. Contact telephone number (Available 24hrs): [redacted].

**Background:**
The research team is trying to help better understand the cartilage changes that occur in knee cartilage after an ACL rupture. You are being contacted because you have been identified from the orthopaedic trauma database at VGH as a patient who has sustained a ruptured ACL.

**Reason for the Study:**
People who have ruptured their ACL are at higher risk of the cartilage in their knees degenerating in the future, that is, developing osteoarthritis. We hope to study whether or not there are early changes in the cartilage properties after an ACL rupture using a special MRI scanner that allows patients to stand up during scans. It has an 'open-to-the-sky' structure and is not a closed tube. Performing MRI scans under weight-bearing conditions gives us potentially a much more realistic understanding of the knee cartilage properties and behaviour.

**Who may Participate:**
We are looking for participants who are:

- Adult participants between the ages of 18-50 years old, with an ACL rupture of only one knee.
- Male and female participants will be equally represented, and we will also seek equal representation of participants who have had their ACL's reconstructed and those that have not.
- Participants must have intact cartilage and evidence of unilateral complete ACL rupture (either from clinical exam or MRI).
- Intact cartilage (to the best of your knowledge).

- Documented unilateral ACL rupture within the last 10 years, reconstructed within 1 year from injury.
- Must have undergone full rehabilitation program and returned to baseline sport/ recreational activities.

If any of the following are true, you **cannot** not participate.

- If you have torn any ligaments OTHER THAN just your ACL (i.e. multiligamentous knee injury: ACL + PCL, LCL, or complete MCL rupture). ACL + incomplete MCL rupture will NOT be excluded. Associated meniscal tear will NOT be excluded.
- Known knee osteoarthritis
- Other joint disease (inflammatory arthritis, prior septic arthritis, osteonecrosis, dysplasia, fracture, or other disease).
- Incompletely rehabilitated injury, defined as range of motion less than 0-130 degrees, visible quads atrophy, or persistent mechanical symptoms during non-sporting activities.
- Staple used in securing one end of the ACL graft, if reconstructed.
- Individuals who cannot undergo MRI (based on MRI screening form); i.e. patients with a cardiac pacemaker or defibrillator, those with metal in their eye or orbit, or a ferromagnetic aneurysm clip, or who are or may be pregnant.
- History of fainting, or orthostatic blood pressure changes of >20mmHg in systolic blood pressure, 10mmHg diastolic blood pressure, or >30 beats per minute change in pulse (this will be checked prior to your scan).
- Prior or subsequent knee surgery other than diagnostic arthroscopy.
- Intra-articular corticosteroid injection to either knee.
- ACL rupture of BOTH knees.
- Re-ruptured ACL.
- Delayed reconstruction of ACL (>1year from injury).

**Voluntary Participation:**
Your participation in this study is entirely voluntary and if you choose not to participate you will not be asked to provide any reason for your choice.

**Study Procedures:**
If you agree to participate, scanning in the UO-MRI will take approximately 3 hours in the morning. The MRI technologist will first complete a detailed MRI Screening Form with you. The scans involve a series of supine (lying down) and upright standing scans.

There is no known or foreseeable risk to your physical health associated with MRI scans. There is a slight risk of claustrophobia (fear associated with confined spaces); however, this is reduced by the open structure of this particular scanner compared to traditional MRI scanners.

There are no direct benefits from participating in this study. You will be able to obtain copies of your scans if you wish, however these scans are for research purposes only and are not clinical scans.

**Remuneration/Reimbursement:**
As this study requires a significant portion of your time, we will reimburse all expenses incurred as a result of your participation. Please bring original travel receipts with you to your scanning session.
We also offer reimbursement of $100 for your time and as appreciation for your participation.

**Contact:**
If you have any questions or desire further information about this study before or during participation, please do not hesitate to contact Dr. David Wilson, [redacted]; he or another member of the study team will be more than happy to respond to all of your questions and concerns.

If you have any concerns or complaints about your rights as a research participant and/or your experiences while participating in this study, contact the Research Participant Complaint Line in the University of British Columbia Office of Research Ethics by e-mail at [redacted] or by phone at [redacted] (Toll Free: [redacted]). Please reference the study number (H18-01459) when calling so the Complaint Line staff can better assist you.

**Confidentiality:**
Your confidentiality will be respected. However, research records and health or other source records identifying you may be inspected in the presence of the Investigator or his or her designate by representatives of the UBC Clinical Research Ethics Board for the purpose of monitoring the research. No information or records that disclose your identity will be released or published without your consent, nor will any information or records that disclose your identity be removed or released without your consent unless required by law. You will be assigned a unique study number as a participant in this study. This number will not include any personal information that could identify you (e.g. it will not include your Personal Health Number, SIN, or your initials, etc.). Only this number will be used on any research-related information collected about you during the course of this study, so that your identity will be kept confidential. Information that contains your identity will remain only with the Principal Investigator and/or designate. The list that matches your name to the unique study number that is used on your research-related information will not be removed or released without your consent unless required by law.

Your rights to privacy are legally protected by federal and provincial laws that require safeguards to ensure that your privacy is respected. You also have the legal right of access to the information about you that has been provided to the sponsor and, if need be, an opportunity to correct any errors in this information. Further details about these laws are available on request to your study doctor.

All of the data collected in this study is confidential. Access to data is restricted to the investigators reported at the opening of this document only. We may also use a completely anonymized and de-identified copy of your MRI scans for educational or promotional purposes, for example: on our website, in a presentation, or in a brochure about our research.

**Participation:**
This letter will be followed up with a phone call by one of the study team members within three weeks of receiving the letter. Or, if you are sure that you want to participate, please e-mail david.stockton@alumni.ubc.ca to schedule a date for your scan, and to receive an official participant consent form.

If you do NOT want any further contact regarding this study, please contact the study co-ordinator at [redacted] or [redacted].

Efforts have been made to ensure this notification does not reach the families of patients who have passed away. If a grieving family member receives this letter, please accept our heartfelt condolences and our sincere apology.

Sincerely,

Dr. Dave Wilson, DPhil
Professor, Dept. of Orthopaedics, UBC
Co-Director, Centre for Hip Health and Mobility

Dr. Pierre Guy, MD, MBA, FRCSC
Associate Professor and Head of Orthopaedic Trauma, UBC
Co-Director, Centre for Hip Health and Mobility

Dr. Bas Masri, MD, FRCSC
Professor and Head, Dept. of Orthopaedics, UBC
and Vancouver Coastal Health

Dr. David Stockton, MD
PGY-5 Orthopaedic Surgery Resident and
UBC Clinician Investigator Program
