# Supplementary material for: Reliability of tibiofemoral contact area and centroid location in upright, open MRI
Source: BMC Musculoskelet Disord. 2020 Nov 30;21:795. doi: 10.1186/s12891-020-03786-1 (PMC7702694; doi:10.1186/s12891-020-03786-1)
Supplement: Supplementary file 2 — Additional file 2. [file 12891_2020_3786_MOESM2_ESM.pdf]

Participant Information and Consent Form – Upright Open MRI

Title: Quantification of cartilage changes using upright, open Magnetic Resonance Imaging (UO-MRI) after Anterior Cruciate Ligament (ACL) injury

Principal Investigator and contact:

Dr. David R. Wilson, DPhil
[redacted]
Department of Orthopaedics, Centre for Hip Health and Mobility, Vancouver Coastal Health Research Institute and University of British Columbia

Co-Investigators:

Dr. Bas A. Masri, MD, FRCSC
Professor and Head, Department of Orthopaedics, UBC
David J. Stockton, BSc, MD
Centre for Hip Health and Mobility, Department of Orthopaedics, UBC
Andrew Yung, MSc
Centre for Hip Health and Mobility, Department of Physics and Astronomy, UBC
Jane Desrochers, PhD
Centre for Hip Health and Mobility, Department of Orthopaedics, UBC
Andrew Schmidt, BSc
Centre for Hip Health and Mobility, Department of Orthopaedics, UBC
Honglin Zhang, PhD
Centre for Hip Health and Mobility, Department of Orthopaedics, UBC

Invitation:
You have been invited to participate in a research study and undergo a magnetic resonance (MR) scan in an open scanner to help us quantify the changes in knee cartilage after sustaining an ACL injury

Purpose:
The purpose of this work is to use the 0.5 Tesla Paramed UO-MRI research scanner, located in the Radiology Department in the Blackmore Pavilion of Vancouver General Hospital, to investigate differences in cartilage loading patterns after ACL injury. The upright, open MRI scanner differs from a traditional MRI scanner in that it is not a closed tube; rather, it has an "open-to-the-sky" structure. This allows much more flexibility in both the types of subjects that may be imaged and also in the position in which the subjects may be imaged.

Voluntary Participation:
Your participation is entirely voluntary, so it is up to you to decide whether or not to take part in this study. Before you decide, it is important for you to understand what the research involves. This consent form will tell you about the study, why the research is being done, what tests will be performed during the study, and the possible benefits, risks and discomforts.

If you wish to participate, you will be asked to sign this form. If you do decide to take part in this study, you are still free to withdraw at any time and without giving any reasons for your decision.

If you do not wish to participate, you do not have to provide any reason for your decision.

Please take time to read the following information carefully and to discuss it with your family, friends, and doctor before you decide.

Participant INCLUSION Criteria:
We are looking for participants who are:

- Adult participants between the ages of 18-50 years old, with unilateral ACL ruptures.
- Male and female participants will be equally represented, and we will also seek equal representation of participants who have had their ACL's reconstructed and those that have not.
- Participants must have intact cartilage and evidence of unilateral complete ACL rupture (either from clinical exam or MRI).
- Intact cartilage (Kellgren-Lawrence grade 0 or 1).
- Documented unilateral ACL rupture within the last 10 years, reconstructed within 1 year from injury.
- Must have undergone full rehabilitation program and returned to baseline sport/ recreational activities.

Participant EXCLUSION Criteria:
If any of the following are true, you cannot not participate.

- If you have torn any ligaments OTHER THAN just your ACL (i.e. multiligamentous knee injury: ACL + PCL, LCL, or complete MCL rupture). ACL + incomplete MCL rupture will NOT be excluded. Associated meniscal tear will NOT be excluded.
- Known knee osteoarthritis (Kellgren-Lawrence grade >1)
- Other joint disease (inflammatory arthritis, prior septic arthritis, osteonecrosis, dysplasia, fracture, or other disease).
- Incompletely rehabilitated injury, defined as range of motion less than 0-130 degrees, visible quads atrophy, or persistent mechanical symptoms during non-sporting activities.
- Staple used in securing one end of the ACL graft, if reconstructed.
- Individuals who cannot undergo MRI (based on MRI screening form); i.e. patients with a cardiac pacemaker or defibrillator, those with metal in their eye or orbit, or a ferromagnetic aneurysm clip, or who are or may be pregnant.
- History of fainting, or orthostatic blood pressure changes of >20mmHg in systolic blood pressure, 10mmHg diastolic blood pressure, or >30 beats per minute change in pulse (this will be checked prior to your scan).
- Prior or subsequent knee surgery other than diagnostic arthroscopy.
- Intra-articular corticosteroid injection to either knee.
- ACL rupture of BOTH knees.

- Re-ruptured ACL.
- Delayed reconstruction of ACL (>1year from injury).

Depending upon the individual situation, you may not be able to participate if you have any of the following:

- artificial heart valve;
- ear or eye implant;
- brain aneurysm clip;
- implanted drug infusion pump;
- electrical stimulator for nerves or bones;
- coil, catheter, or filter in any blood vessel;
- orthopaedic hardware (artificial joint, plate, screw, rod);
- other metallic prostheses;
- shrapnel, bullets or other metal fragments;
- surgery or tattoos (including tattooed eyeliner) in the last six weeks.

If you have any of the above, your individual case will be reviewed by the hospital MR Technologist and/or Radiologist, and a decision will be made regarding your participation in this study. An operative report may be required to assess the nature of the implants in your body.

Study Procedures:
If you choose to participate in this study you will be asked to complete a detailed MR Screening Form asking about contraindications to MRI. This form will be reviewed with you by the MR Technologist to ensure your safety during the scanning session.

You will be asked to change into hospital scrubs and to remove all metal objects (such as hearing aids, dentures, jewellery, watches, hairpins, and ALL piercings) from your body because these objects interfere with imaging and may be attracted to the scanner magnet, or may heat up, with the potential risk of injury. You will be seated in a chair for at least 30 minutes while the screening form and consents are reviewed, to make sure that your knee cartilage is fully unloaded prior to scanning. You will also be given compression socks to be worn while scanning, to minimize venous pooling in your legs. Finally, we will measure your orthostatic blood pressure (blood pressure lying down vs. standing up) to see if you may be at risk of fainting during the upright scans. If there is any evidence that you may be at risk of fainting, you will unfortunately not be able to participate in the study.

You will then be positioned in the MR scanner. This scanner is different from typical MR scanners where subjects are asked to lie on a table which is then moved into the magnet centre (the "doughnut"). The UO-MRI is a vertically open scanner (two parallel discs oriented on edge, 58 cm apart), which allows for a wide range of positioning. An MRI coil (a specifically designed antenna) will be placed near the part of your body we wish to image. The MRI technician will orient the participant to the scanner and instruct them on positioning for the scans, and also on how to use the call bell which is available should the participant feel claustrophobic or faint during scanning. The uninjured knee will be imaged first (as a control), followed by the affected knee with the ACL rupture. Following each scan, the technician will ensure adequate imaging was captured with minimal artifact or motion. The patient will next stand for at least 15 minutes before obtaining the standing scan to allow for loading of knee cartilage to equilibrate. Then standing scans will be obtained, first the uninjured knee followed by the affected knee. Each scan takes approximately 3 minutes. The first 6 participants will be asked to repeat this process again during the same session,

and then asked to return 2 weeks later for more scanning using the same protocol. This data will be used to test the repeatability of the UO-MRI in quantifying knee cartilage loading. Subsequent participants will only attend for one session. Each session will take no longer than 3 hours.

Possible Risks Involved in Participation:
There is no known or foreseeable risk to your physical health associated with MRI scans. There is a slight risk of claustrophobia (fear associated with confined spaces); however, this is reduced by the open structure of this particular scanner as compared to traditional MR scanners. You will be asked to remain as still as possible for the duration of the scanning procedure. During the scan you will hear acoustic noises (very loud "knocking" sounds) from the magnet. You will be required to wear earplugs to minimize the noise. In this scanner, it is possible to image you while you are standing; there is a risk of fainting. Predisposed individuals will be screened out of participation, and precautionary measures will be taken including compression stockings and placing supports and foam mats during scanning. Lastly, a harness 'vest' will be applied to prior to standing scans, to catch you in the rare event that fainting occurs.

Possible Benefits:
You will not receive any direct benefits from participating in this study. You will be able to obtain copies of your scans if you wish, however these scans are for research purposes only and are not clinical scans.

Incidental Findings:
As this will NOT be a medically indicated examination, there will be no formal review of the scans and no report will be made. This MRI scan is not a substitute for one a doctor would order. It may not show problems that would be picked up by a diagnostic MRI scan. However, if we believe that we have found a medical problem in your MRI scan, we will ask a doctor who is trained in the reading of MRI scans, a radiologist, to help us review the images. If the radiologist thinks that there may be an abnormality in your MRI scan that requires follow-up, we will contact you, and with your permission, contact your family physician and help him or her obtain the appropriate follow-up for you. No information generated in this study will become part of your permanent medical record. However, if the study detects an abnormality in your MRI scan and further follow-up is required, then this information may become part of your record.

Confidentiality:
Your confidentiality will be respected. However, research records and health or other source records identifying you may be inspected in the presence of the Investigator or his or her designate by representatives of the UBC Clinical Research Ethics Board for the purpose of monitoring the research. No information or records that disclose your identity will be released or published without your consent, nor will any information or records that disclose your identity be removed or released without your consent unless required by law. You will be assigned a unique study number as a participant in this study. This number will not include any personal information that could identify you (e.g. it will not include your Personal Health Number, SIN, or your initials, etc.). Only this number will be used on any research-related information collected about you during the course of this study, so that your identity will be kept confidential. Information that contains your identity will remain only with the Principal Investigator and/or designate. The list that matches your name to the unique study number that is used on your research-related information will not be removed or released without your consent unless required by law.

Your rights to privacy are legally protected by federal and provincial laws that require safeguards to ensure that your privacy is respected. You also have the legal right of access to the information about you that has been provided to the sponsor and, if need be, an opportunity to correct any errors in this information. Further details about these laws are available on request to your study doctor.

All of the data collected in this study is confidential. Access to data is restricted to the investigators reported at the opening of this document only. We may also use a completely anonymized and de-identified copy of your MRI scans for educational or promotional purposes, for example: on our website, in a presentation, or in a brochure about our research.

Remuneration/Reimbursement:
As this study requires a significant portion of your time, we will reimburse all expenses incurred as a result of your participation. Please bring original receipts with you to your scanning session.
We also offer reimbursement of $100 for your time and as appreciation for your participation.

Refusal or Withdrawal from Study:
This study is strictly voluntary; it is your choice as to whether or not you wish to participate. You may withdraw from this study at any time without giving reasons. If you choose to enter the study and then decide to withdraw at a later time, all information about you collected up to the point of your withdrawal will be retained for analysis in order to protect the integrity of the research, which may benefit future research participants and patients. However, no further information will be collected.

What Happens if Something Goes Wrong?
By signing this form, you do not give up any of your legal rights and you do not release the study doctor, participating institutions, or anyone else from their legal and professional duties. If you become ill or physically injured as a result of participation in this study, medical treatment will be provided at no additional cost to you. The costs of your medical treatment will be paid by your provincial medical plan.

Questions or Concerns Regarding the Study:
If you have any questions or desire further information about this study before or during participation, please do not hesitate to contact Dr. David Wilson, [redacted]; he or another member of the study team will be more than happy to respond to all of your questions and concerns.

If you have any concerns or complaints about your rights as a research participant and/or your experiences while participating in this study, contact the Research Participant Complaint Line in the University of British Columbia Office of Research Ethics by e-mail at [redacted] or by phone at [redacted] (Toll Free: [redacted]). Please reference the study number (H18-01459) when calling so the Complaint Line staff can better assist you.

Consent:
I have read and understood all of the statements above. I realize that participation in this study is strictly voluntary and that I may refuse to participate or I may withdraw at any time. I recognize that all of the measurements are in addition to my normal health care. I understand that I am not waiving my legal rights by signing this consent form.

I have been told that I will receive a **signed and dated** copy of this document for my personal records.

By signing this document, I consent to participate in this study.

☐ Please check this box if you wish to have your family doctor contacted in the case that there are incidental findings.

Participant ______________________ Date ______________________
(please print)

Signature______________________

Witness______________________ Date ______________________
(please print)

Signature______________________

Investigator______________________ Date ______________________
(please print)

Signature______________________

Please provide the following information so that we may contact you in the case that there are incidental findings in your scan. Only include your family doctor's name if you would like them to also be contacted regarding any incidental findings.

PLEASE PRINT

Name______________________________________

Phone:______________________________________

Date of Birth:
(dd/mm/yy)__________________________________

Family Doctor:________________________________
