# Supplementary material for: Reliability of tibiofemoral contact area and centroid location in upright, open MRI
Source: BMC Musculoskelet Disord. 2020 Nov 30;21:795. doi: 10.1186/s12891-020-03786-1 (PMC7702694; doi:10.1186/s12891-020-03786-1)
Supplement: Supplementary file 3 — Additional file 3. [file 12891_2020_3786_MOESM3_ESM.pdf]

CENTRE for HIP Health and Mobility

How to Prepare for your Upright Open MRI Standing Scan

Standing MRI scans provide a unique way to look at the human body in clinically relevant weight-bearing positions. Some people feel lightheaded or faint during standing scans. To reduce this possibility we ask that you follow these preparation instructions:

Before arriving for your scan:

- **Eat a healthy full meal before coming to your scanning appointment**
- Increase your fluid consumption while limiting the amount of caffeine (compared to your normal routine)
- You may otherwise carry on with your normal routine (e.g. exercise, etc.) but please be cognisant of your body's nutritional and fluid needs
- Plan to have something to drink and eat just prior to your scan, e.g. water or juice, and a piece of fruit, granola bar, or other snack.

What you can expect during your scan:

- You will be given a call bell to squeeze if at any time you feel faint, dizzy, lightheaded or otherwise unwell
- You will be given juice and a granola bar approximately 30 minutes prior to the standing portion of your scan (whenever there is an appropriate pause between scans)
- You will be given a short rest period between standing scans (each scan is typically less than 5 minutes in length), during which we will ask you to flex your legs, squat, or otherwise increase blood flow in your legs
- You will wear fabric compression leggings around your calves that will gently squeeze your calves to increase blood flow to and from your legs
- You will be given bathroom breaks as needed
- During standing scans, it is very important that you DO NOT LOCK YOUR KNEES, but rather keep your legs comfortably straight with a slight bend at the knee as per your normal posture. Locking your knees restricts the return of venous blood to your heart and brain.

When you arrive at the scanner:

In addition to the MRI Safety Screening, we will ask you questions about relevant health history. Please check all that apply and bring this sheet to your appointment:

- ☐ Do you have epilepsy?
- ☐ Have you fainted in the past? If yes, please describe the situation(s)
- ☐ Do you have low blood pressure?
- ☐ Are you hypoglycemic?
- ☐ Are you anemic?
- ☐ In the past week have you donated blood?
- ☐ In the past two weeks have you been ill (cold, flu, etc.)?
- ☐ Do you have circulatory problems in your legs?
- ☐ Is there any other reason that you might be at increased risk of fainting?

We look forward to having you participate in Upright Open MRI Research!

Vancouver Coastal Health Authority

☐ LGH ☐ RH ☐ UBCH ☐ VGH

MAGNETIC RESONANCE IMAGING (MRI) PATIENT SCREENING FORM

STUDY ID: ______________________

DATE: ______________________

Every patient scheduled for MRI **MUST** complete the following questionnaire prior to the being scanned. The technologist will be happy to answer any of your questions. **Please answer each question accurately and explain any marked "yes".**

Birth date: _____________ Age: _______ Height____ft____in or ____cm Weight:______kg or _______lbs

Do you have:	Yes	No	Unsure	If yes, explain
Cardiac (Heart) Pacemaker or Wires (At any time in your life)				
Artificial Heart Valves				
Brain aneurysm clips				
Metal in your eyes (At any time in your life)				
Implanted Electrodes, Pumps or Catheters				
Neurostimulators				
Shrapnel, Bullets or other metal fragments				
Any Tattoos – including permanent make up				
Ear implants (Cochlear, Stapes) /Hearing Aid				
Orthopedic (Bone) Screws, Pins, Plates, Rods (If yes, state location)				
Breast tissue expander or other implants				
Prosthesis (Eye, Penile, Leg, Arm, Joint, etc.)				
Any Stents, Coils, or Filter in blood vessels				
Dentures, retainer, braces, magnetic implants				
Transdermal medication patches (Examples: Nitroglycerin for heart or Nicotine to stop smoking)				
Body Piercing other than earrings				
**Have you ever had surgery or operation on:**				
Brain, Eye, or Ear				
Heart				
Neck, Chest, or Back (Spine)				
Abdomen, Pelvis, Hips				
Arms and/or Legs				
Injection into a joint within the last 2 weeks				
**Are you:**				
Pregnant				
Claustrophobic				

Please remove all your jewelry, watch, credit cards, coins and other metallic items (earrings, hair clips, bobby pins, etc.). A MRI staff member will instruct you about securing your items prior to entry into the examination area. I have read and understand the entire contents of this form. I affirm that the above information is true to the best of my knowledge and I hereby consent to the MRI study

______________________ Signature of person completing this form — ______________ Date

______________________ Relationship to patient if form not completed by patient — ______________ Review Date — ______________ Patient Initials

______________________ Signature of translator — ______________ Date

MR Technologist Initials/Date [ ]

If your MRI exam date occurs after the date the screening form was completed, you must review the screening form and alert the MR technologist of any changes. Please enter the date of review and your initials indicating confirmation of review.
