# Supplementary material for: Reliability of tibiofemoral contact area and centroid location in upright, open MRI
Source: BMC Musculoskelet Disord. 2020 Nov 30;21:795. doi: 10.1186/s12891-020-03786-1 (PMC7702694; doi:10.1186/s12891-020-03786-1)
Supplement: Supplementary file 4 — Additional file 4. [file 12891_2020_3786_MOESM4_ESM.pdf]

Supplementary Table A

Intra-Rater Contact Area Values: Sagittal								
Subject	Segmentation	CA-Med (mm^2)	CA Med SEM (mm^2)	CA-Lat (mm^2)	CA Lat SEM (mm^2)	Tibial Plateau Area (mm^2)	Normalized CA-Med	Normalized CA-Lat
1	1	498.80	3.99	333.25	2.15	3693.56	13.51	9.02
	2	496.65		333.25			13.45	9.02
	3	485.90		339.70			13.16	9.20
2	1	258.00	4.48	292.40	3.58	2977.63	8.67	9.82
	2	273.05		303.15			9.20	10.18
	3	268.75		303.15			9.03	10.18
3	1	412.80	5.87	356.90	10.11	3096.71	13.33	11.53
	2	427.85		352.60			13.82	11.39
	3	432.15		384.85			13.96	12.43
4	1	513.85	8.81	245.10	11.84	3266.81	15.73	7.50
	2	533.20		227.90			16.32	6.98
	3	543.95		268.75			16.65	8.23
5	1	546.10	9.32	311.75	11.93	3311.19	16.49	9.42
	2	531.05		285.95			16.04	8.64
	3	563.30		270.90			17.01	8.18
**Averages**		**N/A**	**6.49**	**N/A**	**7.92**	**3269.18**	**N/A**	**N/A**

Supplementary Table B

Intra-Rater Contact Area Values: Coronal								
**Subject**	**Segmentation**	**CA-Med (mm2)**	**CA Med SEM (mm2)**	**CA-Lat (mm2)**	**CA Lat SEM (mm2)**	**Tibial Plateau Area (mm2)**	**Normalized CA-Med**	**Normalized CA-Lat**
1	1	445.05	10.85	324.65	12.18	3693.56	12.05	8.80
	2	470.85		288.10			12.75	7.80
	3	481.60		288.10			13.04	7.80
2	1	277.35	4.70	281.65	1.90	2977.63	9.31	9.46
	2	262.30		288.10			8.81	9.68
	3	275.20		283.80			9.24	9.53
3	1	455.80	9.69	301.00	3.12	3096.71	14.72	9.72
	2	425.70		290.25			13.75	9.37
	3	427.85		294.55			13.82	9.51
4	1	483.75	12.68	191.35	3.79	3266.81	14.81	5.86
	2	524.60		204.25			16.06	6.25
	3	518.15		199.95			15.86	6.12
5	1	537.50	8.08	328.95	11.38	3311.19	16.23	9.93
	2	524.60		303.15			15.84	9.16
	3	509.55		290.25			15.39	8.77
**Averages**		**N/A**	**9.20**	**N/A**	**6.47**	**3269.18**	**N/A**	**N/A**

Supplementary Table C

Intra-Rater Centroid Positions Normalized: Sagittal					
**Subject**	**Segmentation**	**Med X (Med-Lat %)**	**Med Y (Post-Ant %)**	**Lat X (Med-Lat %)**	**Lat Y (Post-Ant %)**
1	1	23.25	66.17	71.30	47.48
	2	22.86	67.45	72.01	47.35
	3	23.71	66.17	71.02	45.94
2	1	27.15	65.76	72.90	29.99
	2	26.91	71.48	73.10	31.03
	3	27.19	71.61	73.69	31.47
3	1	23.75	60.14	70.70	48.46
	2	24.01	61.82	72.09	47.79
	3	24.71	61.11	72.03	47.74
4	1	28.27	60.47	76.55	63.42
	2	28.21	49.19	76.50	62.64
	3	28.98	48.93	76.43	62.47
5	1	24.75	50.71	72.67	40.00
	2	25.03	52.00	71.78	38.52
	3	25.08	54.46	72.25	41.19

Supplementary Table D

Intra-Rater Centroid Positions Normalized: Coronal					
**Subject**	**Segmentation**	**Med X (Med-Lat %)**	**Med Y (Post-Ant %)**	**Lat X (Med-Lat %)**	**Lat Y (Post-Ant %)**
1	1	23.06	65.61	73.79	54.00
	2	23.84	66.21	74.15	51.00
	3	23.49	66.22	74.00	50.65
2	1	25.63	66.99	73.66	28.47
	2	26.11	67.26	73.46	29.75
	3	25.76	67.41	73.50	30.27
3	1	24.30	57.92	71.61	49.07
	2	23.43	58.76	71.08	49.76
	3	23.73	57.89	71.14	48.82
4	1	26.39	59.25	74.27	52.37
	2	26.95	59.74	75.28	52.78
	3	27.10	59.67	75.44	52.42
5	1	20.64	57.90	68.85	55.05
	2	21.56	60.52	68.71	55.09
	3	21.96	61.95	68.94	54.71

Supplementary Table E

Intra-Rater Centroid Positions Absolute: Sagittal									
**Subject**	**Segmentation**	**Med X (Med-Lat mm)**	**Med X SEM (mm)**	**Med Y (Post-Ant mm)**	**Med Y SEM (mm)**	**Lat X (Med-Lat mm)**	**Lat X SEM (mm)**	**Lat Y (Post-Ant mm)**	**Lat Y SEM (mm)**
1	1	17.40	0.18	35.85	0.23	53.35	0.22	25.72	0.27
	2	17.10		36.54		53.88		25.66	
	3	17.74		35.85		53.14		24.89	
2	1	18.45	0.06	30.54	0.90	49.53	0.16	13.93	0.20
	2	18.29		33.19		49.67		14.41	
	3	18.47		33.25		50.07		14.62	
3	1	17.36	0.21	27.41	0.22	51.68	0.33	22.09	0.11
	2	17.55		28.18		52.70		21.78	
	3	18.06		27.85		52.65		21.76	
4	1	20.18	0.18	29.12	1.83	54.64	0.02	30.54	0.14
	2	20.14		23.69		54.61		30.17	
	3	20.69		23.56		54.56		30.09	
5	1	17.24	0.07	27.92	0.61	50.62	0.18	22.02	0.42
	2	17.44		28.63		50.01		21.21	
	3	17.47		29.98		50.33		22.68	
**Averages**		**N/A**	**0.14**	**N/A**	**0.76**	**N/A**	**0.18**	**N/A**	**0.23**

Supplementary Table F

Intra-Rater Centroid Positions Absolute: Coronal									
**Subject**	**Segmentation**	**Med X (Med-Lat mm)**	**Med X SEM (mm)**	**Med Y (Post-Ant mm)**	**Med Y SEM (mm)**	**Lat X (Med-Lat mm)**	**Lat X SEM (mm)**	**Lat Y (Post-Ant mm)**	**Lat Y SEM (mm)**
1	1	17.26	0.17	35.55	0.11	55.21	0.08	29.26	0.58
	2	17.84		35.87		55.48		27.63	
	3	17.57		35.88		55.37		27.44	
2	1	17.41	0.10	31.11	0.06	50.05	0.04	13.22	0.25
	2	17.74		31.23		49.91		13.82	
	3	17.50		31.31		49.93		14.06	
3	1	17.76	0.18	26.40	0.13	52.35	0.12	22.37	0.13
	2	17.13		26.78		51.96		22.68	
	3	17.35		26.39		52.00		22.25	
4	1	18.84	0.15	28.53	0.07	53.01	0.26	25.22	0.06
	2	19.24		28.77		53.74		25.42	
	3	19.35		28.74		53.85		25.25	
5	1	14.37	0.27	31.88	0.65	47.96	0.05	30.30	0.07
	2	15.02		33.32		47.87		30.33	
	3	15.30		34.11		48.03		30.12	
**Averages**		**N/A**	**0.18**	**N/A**	**0.20**	**N/A**	**0.11**	**N/A**	**0.22**

Supplementary Table G

Inter-Rater Contact Area Values: Sagittal								
**Subject**	**Rater**	**CA-Med (mm^2)**	**CA Med SEM (mm^2)**	**CA-Lat (mm^2)**	**CA Lat SEM (mm^2)**	**Tibial Plateau Area (mm^2)**	**Normalized CA-Med**	**Normalized CA-Lat**
1	1	498.80	24.73	333.25	15.05	3693.56	13.51	9.02
	2	548.25		303.15			14.83	8.20
2	1	258.00	25.80	292.40	19.35	2977.63	8.67	9.82
	2	309.60		331.10			10.40	11.12
3	1	412.80	0.00	356.90	18.28	3096.71	13.33	11.53
	2	412.80		393.45			13.33	12.71
4	1	513.85	30.10	245.10	23.65	3266.81	15.73	7.50
	2	574.05		292.40			17.57	8.95
5	1	494.50	3.22	296.70	1.08	3311.19	14.93	8.96
	2	500.95		298.85			15.13	9.03
**Averages**		**N/A**	**16.77**	**N/A**	**15.48**	**3269.18**	**N/A**	**N/A**

Supplementary Table H

Inter-Rater Contact Area Values: Coronal								
**Subject**	**Rater**	**CA-Med (mm^2)**	**CA Med SEM (mm^2)**	**CA-Lat (mm^2)**	**CA Lat SEM (mm^2)**	**Tibial Plateau Area (mm^2)**	**Normalized CA-Med**	**Normalized CA-Lat**
1	1	445.05	20.43	324.65	26.88	3693.56	12.05	8.79
	2	485.90		270.90			13.16	7.33
2	1	277.35	21.50	281.65	8.60	2977.63	9.31	9.46
	2	234.35		264.45			7.87	8.88
3	1	455.80	2.15	301.00	18.28	3096.71	14.72	9.72
	2	460.10		264.45			14.86	8.54
4	1	483.75	24.73	191.35	4.30	3266.81	14.81	5.86
	2	434.30		182.75			13.29	5.59
5	1	535.35	25.80	277.35	7.52	3311.19	16.17	8.38
	2	483.75		292.40			14.61	8.83
**Averages**		**N/A**	**18.92**	**N/A**	**13.12**	**3269.18**	**N/A**	**N/A**

Supplementary Table I

Inter-Rater Centroid Positions Normalized: Sagittal					
**Subject**	**Rater**	**Med X (Med-Lat %)**	**Med Y (Post-Ant %)**	**Lat X (Med-Lat %)**	**Lat Y (Post-Ant %)**
1	1	23.25	66.17	71.30	47.48
	2	24.40	66.00	71.84	48.33
2	1	26.91	71.48	73.10	31.03
	2	27.15	65.76	72.90	29.99
3	1	23.75	60.14	70.70	48.46
	2	25.49	63.51	72.94	42.07
4	1	28.27	60.47	76.55	63.42
	2	25.88	61.91	73.48	49.69
5	1	24.09	68.88	67.89	39.30
	2	24.71	71.49	69.77	40.33

Supplementary Table J

Inter-Rater Centroid Positions Normalized: Coronal					
**Subject**	**Rater**	**Med X (Med-Lat %)**	**Med Y (Post-Ant %)**	**Lat X (Med-Lat %)**	**Lat Y (Post-Ant %)**
1	1	23.06	65.61	73.79	54.00
	2	24.00	58.95	72.94	48.30
2	1	25.63	66.99	73.66	28.47
	2	26.35	67.02	73.81	29.25
3	1	24.30	57.92	71.61	49.07
	2	25.46	60.41	68.13	46.92
4	1	26.39	59.25	74.27	52.37
	2	26.04	58.95	71.26	54.09
5	1	26.84	75.09	72.67	45.98
	2	28.06	75.14	72.01	47.70

Supplementary Table K

Inter-Rater Centroid Positions Absolute: Sagittal									
**Subject**	**Segmentation**	**Med X (Med-Lat mm)**	**Med X SEM (mm)**	**Med Y (Post-Ant mm)**	**Med Y SEM (mm)**	**Lat X (Med-Lat mm)**	**Lat X SEM (mm)**	**Lat Y (Post-Ant mm)**	**Lat Y SEM (mm)**
1	1	17.40	0.43	35.85	0.05	53.35	0.20	25.72	0.23
	2	18.26		35.76		53.75		26.18	
2	1	18.29	0.08	33.19	1.33	49.67	0.07	14.41	0.24
	2	18.45		30.54		49.53		13.93	
3	1	17.36	0.64	27.41	0.77	51.68	0.82	22.09	1.46
	2	18.64		28.95		53.32		19.18	
4	1	20.18	0.85	29.12	0.35	54.64	1.09	30.54	3.31
	2	18.48		29.82		52.45		23.93	
5	1	16.78	0.22	37.92	0.72	47.29	0.66	21.63	0.28
	2	17.21		39.36		48.60		22.20	
**Averages**		**N/A**	**0.44**	**N/A**	**0.64**	**N/A**	**0.57**	**N/A**	**1.10**

Supplementary Table L

Inter-Rater Centroid Positions Absolute: Coronal									
**Subject**	**Segmentation**	**Med X (Med-Lat mm)**	**Med X SEM (mm)**	**Med Y (Post-Ant mm)**	**Med Y SEM (mm)**	**Lat X (Med-Lat mm)**	**Lat X SEM (mm)**	**Lat Y Pos (Post-Ant mm)**	**Lat Y SEM (mm)**
1	1	17.26	0.35	35.55	1.81	55.21	0.32	29.26	1.54
	2	17.96		31.94		54.58		26.17	
2	1	17.41	0.24	31.11	0.01	50.05	0.05	13.22	0.18
	2	17.90		31.12		50.15		13.58	
3	1	17.76	0.43	26.40	0.57	52.35	1.27	22.37	0.49
	2	18.61		27.54		49.80		21.38	
4	1	18.84	0.12	28.53	0.07	53.01	1.07	25.22	0.41
	2	18.59		28.39		50.86		26.05	
5	1	18.70	0.42	41.34	0.01	50.62	0.23	25.31	0.47
	2	19.54		41.36		50.16		26.26	
**Averages**		**N/A**	**0.31**	**N/A**	**0.49**	**N/A**	**0.59**	**N/A**	**0.62**

Supplementary Table M

Test-Retest Contact Area Values: Sagittal								
**Subject**	**Scan Session**	**CA-Med (mm^2)**	**CA Med SEM (mm^2)**	**CA-Lat (mm^2)**	**CA Lat SEM (mm^2)**	**Tibial Plateau Area (mm^2)**	**Normalized CA-Med**	**Normalized CA-Lat**
1	1	498.80	7.53	333.25	11.83	3693.56	13.51	9.02
	2	513.85		356.90			13.91	9.66
2	1	258.00	9.68	292.40	15.05	2977.63	8.67	9.82
	2	277.35		322.50			9.31	10.83
3	1	412.80	15.05	356.90	10.75	3096.71	13.33	11.53
	2	442.90		335.40			14.3	10.83
4	1	513.85	8.60	245.10	11.83	3266.81	15.73	7.50
	2	496.65		268.75			15.20	8.22
5	1	546.10	25.80	311.75	7.53	3311.19	16.49	9.42
	2	494.50		296.70			14.93	8.96
**Averages**		**N/A**	**13.33**	**N/A**	**11.40**	**3269.18**	**N/A**	**N/A**

Supplementary Table N

Test-Retest Contact Area Values: Coronal								
**Subject**	**Scan Session**	**CA-Med (mm^2)**	**CA Med SEM (mm^2)**	**CA-Lat (mm^2)**	**CA Lat SEM (mm^2)**	**Tibial Plateau Area (mm^2)**	**Normalized CA-Med**	**Normalized CA-Lat**
1	1	445.05	4.30	324.65	8.60	3693.56	12.05	8.79
	2	436.45		307.45			11.82	8.32
2	1	277.35	4.30	281.65	2.15	2977.63	9.31	9.46
	2	285.95		285.95			9.60	9.60
3	1	455.80	4.30	301.00	11.83	3096.71	14.72	9.72
	2	447.20		324.65			14.44	10.48
4	1	483.75	16.13	191.35	22.57	3266.81	14.81	5.86
	2	516.00		236.50			15.80	7.24
5	1	537.50	1.07	328.95	25.80	3311.19	16.23	9.93
	2	535.35		277.35			16.17	8.38
**Averages**		**N/A**	**6.02**	**N/A**	**14.19**	**3269.18**	**N/A**	**N/A**

Supplementary Table O

Test-Retest Centroid Positions Normalized: Sagittal					
**Subject**	**Scan Session**	**Med Centroid X (Med-Lat %)**	**Med Centroid Y (Post-Ant %)**	**Lat Centroid X (Med-Lat %)**	**Lat Centroid Y (Post-Ant %)**
1	1	23.25	66.17	71.30	47.48
	2	24.22	63.27	69.01	53.54
2	1	27.15	65.76	72.90	29.99
	2	26.94	61.77	73.49	30.70
3	1	23.75	60.14	70.70	48.46
	2	27.49	62.95	74.20	47.82
4	1	28.27	60.47	76.55	63.42
	2	24.40	65.78	71.05	61.68
5	1	24.75	50.71	72.67	40.00
	2	24.09	48.88	67.89	39.30

Supplementary Table P

Test-Retest Centroid Positions Normalized: Coronal					
**Subject**	**Scan Session**	**Med Centroid X (Med-Lat %)**	**Med Centroid Y (Post-Ant %)**	**Lat Centroid X (Med-Lat %)**	**Lat Centroid Y (Post-Ant %)**
1	1	23.06	65.61	73.79	54.00
	2	23.32	61.72	70.87	58.36
2	1	25.63	66.99	73.66	28.47
	2	24.00	68.67	70.74	33.14
3	1	24.30	57.92	71.61	49.07
	2	29.00	64.48	73.00	48.23
4	1	26.39	59.25	74.27	52.37
	2	27.01	65.97	74.41	64.80
5	1	20.64	57.90	68.85	55.05
	2	26.84	55.09	72.67	45.98

Supplementary Table Q

Test-Retest Centroid Positions Absolute: Sagittal									
**Subject**	**Scan session**	**Med X (Med-Lat mm)**	**Med X SEM (mm)**	**Med Y (Post-Ant mm)**	**Med Y SEM (mm)**	**Lat X (Med-Lat mm)**	**Lat X SEM (mm)**	**Lat Y (Post-Ant mm)**	**Lat Y SEM (mm)**
1	1	17.40	0.36	35.85	0.78	53.35	0.86	25.72	1.64
	2	18.12		34.28		51.64		29.01	
2	1	18.45	0.07	30.54	0.93	49.53	0.20	13.93	0.16
	2	18.30		28.69		49.93		14.26	
3	1	17.36	1.36	27.41	0.64	51.68	1.28	22.09	0.15
	2	20.09		28.69		54.24		21.80	
4	1	20.18	1.38	29.12	1.28	54.64	1.96	30.54	0.42
	2	17.42		31.68		50.71		29.71	
5	1	17.24	0.23	27.92	0.50	50.62	1.67	22.02	0.19
	2	16.78		26.91		47.29		21.63	
**Averages**		**N/A**	**0.68**	**N/A**	**0.83**	**N/A**	**1.19**	**N/A**	**0.51**

Supplementary Table R

Test-Retest Centroid Positions Absolute: Coronal									
**Subject**	**Scan session**	**Med X (Med-Lat mm)**	**Med X SEM (mm)**	**Med Y (Post-Ant mm)**	**Med Y SEM (mm)**	**Lat X (Med-Lat mm)**	**Lat X SEM (mm)**	**Lat Y (Post-Ant mm)**	**Lat Y SEM (mm)**
1	1	17.26	0.10	35.55	1.06	55.21	1.09	29.26	1.18
	2	17.45		33.44		53.03		31.62	
2	1	17.41	0.55	31.11	0.39	50.05	0.99	13.22	1.08
	2	16.31		31.89		48.06		15.39	
3	1	17.76	1.72	26.40	1.50	52.35	0.51	22.37	0.19
	2	21.20		29.39		53.36		21.98	
4	1	18.84	0.22	28.53	1.62	53.01	0.05	25.22	2.99
	2	19.28		31.77		53.11		31.21	
5	1	14.37	2.16	31.88	0.77	47.96	1.33	30.30	2.49
	2	18.70		30.33		50.62		25.31	
**Averages**		**N/A**	**0.95**	**N/A**	**1.07**	**N/A**	**0.79**	**N/A**	**1.59**

Supplementary Table S

Bovine Tibiofemoral Sample Accuracy			
	**CA- 7T Bruker (mm^2)**	**CA- 0.5T MROpen (mm^2)**	**Difference (mm^2)**
Medial Unloaded	N/A	N/A	N/A
Medial Loaded	125.69	120.40	5.29
Lateral Unloaded	258.43	270.9	12.47
Lateral Loaded	239.56	253.7	14.14
**Mean Absolute Error**			**10.63**
